# Primary total hip arthroplasty using an uncemented Wagner SL stem in elderly patients with Dorr type C femoral bone

**DOI:** 10.1186/s13018-019-1421-5

**Published:** 2019-11-21

**Authors:** Ping Zhen, Jun Liu, Xusheng Li, Hao Lu, Shenghu Zhou

**Affiliations:** 1 Department of Orthopedics, The Second Affiliated Hospital of Lanzhou University, Cuiying Door No. 82, Chengguan District, Lanzhou City, 730030 Gansu Province People’s Republic of China; 2 Department of Joint Surgery, Institute of Orthopedics, The 940th Hospital of PLA Joint Logistics Support Force, South Binhe Road, No. 333, Lanzhou City, 730050 Gansu Province People’s Republic of China

**Keywords:** Primary total hip arthroplasty, Cementless, Wagner SL stem, Type C femoral bone

## Abstract

**Background:**

The purpose of this study was to review retrospectively the primary total hip arthroplasties operated upon with the cementless Wagner Self-Locking stem in patients with type C femoral bone.

**Methods:**

Twenty-eight total hip arthroplasties were performed in 25 patients aged ≥ 60 years using a cementless Wagner Self-Locking femoral component between 2006 and 2011. According to Dorr’s criteria, all 28 femora were classified as type C bone. All patients were treated with THA using a cementless Wagner cone prosthesis. Clinical and radiologic evaluations were performed on all patients.

**Results:**

Mean follow-up period was 125 ± 10.5 months (range 96 to 156 months). Average Harris hip score pre-operatively was 46 ± 9 (range 39 to 62) and at the last follow-up was 90 ± 9 (range 83 to 98). The stem to canal fill is calculated as percentages on the operative side at three distinct levels: just below the lesser trochanter, at midstem, and 1 cm above the tip of the component on anteroposterior radiograph. The mean proximal stem-to-canal fill percentages were 97% ± 2.1%, 95% ± 3.5%, and 88% ± 2.6%, respectively (anteroposterior view) and 92% ± 2.2%, 86% ± 1.9%, and 83% ± 2.5%, respectively (lateral view). Radiographic evaluation demonstrated good osteointegration of the implants in the follow-up.

**Conclusions:**

Based on the long-straight cylindrical tapered stem design, the cementless Wagner SL stem can achieve reliable stability by close apposition of the stem and wide stovepipe femoral canal from metaphysis to diaphysis in type C bone.

## Introduction

As described by Dorr et al. [1], type C femoral bone, characterized by wide, patulous canals with poor bone quality, always presents a challenge for fixation of cementless implants in patients undergoing total hip arthroplasty [2–6]. Type C bone is relatively common among the elderly population, especially in older postmenopausal females [1, 2]. Patients with Dorr type C bone always exhibit wide, stovepipe-shaped femoral canals, and thin cortices in proximal femur [3]. The combination of abnormal bone shape and a presumed poorer local biologic environment are the reasons that surgeons have been hesitant to use uncemented prosthesis in these femora [4–6]. Traditionally, femoral fixation in these patients had been obtained with polymethylmethacrylate bone cement [2, 5, 7]. However, the long-term performance of cemented stems may be compromised by fixation loosening, osteolysis, and revision problems [5]. For the past 10 years, more modern techniques have been developed and more advanced formal implant designs have become available [4–6], which have prompted to look at cementless reconstruction of the femur in presence of unusual proximal femoral anatomy [8–10]. Nevertheless, a mismatch between the geometry of the most current femoral stems and abnormal morphology of proximal femoral canals is the greatest hindrance to the use of cementless stems in type C bone although some results of conventional large diameter or tapered cementless stems are generally good in those patients [4–6, 9, 10]. In fact, most cementless primary stems cannot achieve an anatomic fit and fill from the diaphysis to metaphysis in an extremely wide stovepipe-shaped femoral canal [4–7].

The Wagner SL implant is a long-straight stem femoral prosthesis with tapered geometry and raised ridges that are designed for revision. This prosthesis was originally designed by Wagner in 1987 [11] for treating patients with severe bone loss in their proximal femurs [12]. The clinical efficacy using the Wagner SL prosthesis has been widely publicized in revision surgery [11–13]. The third generation of Wagner SL femoral stem occupies cylindrical straight tapered shape and long straight shaft which is suitable to be used not only in the extensive metadiaphyseal deficiency and a widened femoral canal in revision but also in the stovepipe endosteal canal in type C bone.

On the basis of these available data, it was our hypothesis that this prosthesis stem would allow for stable biologic fixation in a wide spectrum of femoral geometry, including younger or older patients with wider femoral canals in primary total hip arthroplasty. Furthermore, we also theorized that close geometrical matching between the cementless stem and femoral canal was the key factor obtaining intimate diaphyseal fit, fill, and biologic fixation.

In the present study, we extended the use of cementless Wagner SL stems to primary total hip arthroplasty in patients with type C bone. The aim of the current study was to analyze the possibilities of the cementless Wagner SL stem for primary total hip arthroplasty (THA) in type C bone. Experience with geometric match between the Wagner SL stem and widened stovepipe femoral canal was of special interest.

## Material and methods

### Femoral stem

The Wagner self-locking stem (Zimmer, Warsaw, IN, USA) is made of a biocompatible TiAlNb alloy with an extensive grit-blasted titanium surface. The third generation of Wagner SL stem is designed to a long-straight stem with cylindrical tapered geometry [12]. The stem is available in lengths of 190 to 305 mm and diameters of 14 to 25 mm. The 2° conical design of the stem with the eight longitudinal ribs provides good rotational stability in the femoral diaphysis. A grit-blasted titanium surface provides a good press-fit and promotes bone ongrowth [13].

### Patient demographics

A retrospective study including 28 hips in 25 consecutive patients with type C femoral bone of Dorr classification [1] was performed. All patients underwent primary total hip arthroplasty using an uncemented Wagner SL Stem (Zimmer®) between January 2006 and January 2011. All operations were performed by two senior surgeons. This study conforms to the Declaration of Helsinki as revised in 2008 and was authorized by the ethical committee of the authors’ institution. All patients gave informed consent to this study.

There were 22 females (25 hips) and 3 males (3 hips). The average age at the time of surgery was 72.5 ± 5.3 years (range 60.3 to 82.6 years). The patients’ mean height, body weight, and body mass index were 162.4 ± 3.2 cm (range 155.8 to 174.3 cm), 65.3 ± 6.7 kg (range 58.5 to 81.1 kg), and 27.3 ± 2.8 (range 21.8 to 33.8), respectively. The original diagnosis was primary osteoarthritis in 5 hips (5 patients), rheumatoid arthritis in 15 hips (18 patients), and ankylosing spondylitis in 5 hips (5 patients). All 25 patients were living independently before admission.

Preoperatively, all patients were evaluated clinically and radiographically. For each patient, the complete medical history was collected. Pain and grade of disability were assessed in terms of limitation of the hip range of motion and restrictions on walking daily activities. Clinical evaluation was rated with the Harris hip score [14]. For all patients, standard radiographs of the pelvis in anteroposterior view and of the affected hip in axial view were obtained. On radiographs, femoral bone status was ascertained before the operation and the appropriate implant was chosen by preoperative templating (see Table 1 patient demographics).

**Table 1:** Patient demographics

Demographics	Data
Gender	22 females(25 hips), 3 males(3 hips)
Mean age	72.5 ± 5.3 years (range 60.3 to 82.6 years)
Height	162.4 ± 3.2 cm (range 155.8 to 174.3 cm)
Weight	65.3 ± 6.7 kg (range 58.5 to 81.1 kg)
BMI	27.3 ± 2.8 (range 21.8 to 33.8)
Disease classification	Osteoarthritis in 5 patients(5 hips), rheumatoid arthritis in 13 patients(15 hips), ankylosing spondylitis in 5 patient (5 hips)
Dorr index	0.75 ± 0.31 (range 0.68 to 0.95)
Femoral canal dimension	18.5 ± 2.9 mm (range 16.2 to 22.7 mm)
Cortical index	0.37 ± 0.23 (range 0.21 to 0.48)
Follow-up time	72 ± 8.5 months (range 57 to 85 months)
Preoperative Harris score	46 ± 9 (range 39 to 62)
12 months after surgery of Harris score	90 ± 9 (range 83 to 98)
Last follow-up of Harris score	90 ± 9 (range 83 to 98)
Stem position	Neutral alignment (19), varus position (5), valgus position (1)
Proximal stem-to-canal fill percentage	95% ± 3.5% (anteroposterior view), 90% ± 2.8% (lateral view)
Distal canal fill percentage	93% ± 4.5% (anteroposterior), 92% ± 3. 9% (lateral)
Bone resorption and stress shielding	5 hips (grade 1 in 3 hips, grade 2 in 2 hips)
Bone-prosthetic landmarks	2 stem (2 mm), 1 stem (3 mm)

### Radiographic evaluation

The femoral morphology was classified according to Dorr et al. [1]. The canal-to-calcar ratio (CC ratio) was calculated as the fraction of the isthmus canal width divided by the calcar canal dimension. According to the Dorr classification, a CC ratio of > 0.64 is considered to indicate type C bone [1]. The transverse diameter of the medullary canal and the thickness of the cortex of the femur were also assessed. The femoral canal dimension at the isthmus was measured on the anteroposterior view of the pelvis and the cortical index is a quotient calculated as the thickness of the cortex divided by the diameter of the femur 10 cm from the midpoint of the lesser trochanter [15]. In this study, femoral bone morphology was classified as Dorr type C bone in all 28 hips of (described as having a stovepipe shape with a wide femoral canal and thin cortices). In the anteroposterior view, the average CC ratio in these 28 hips (25 patients) was 0.75 ± 0.31 (range 0.68 to 0.95), the average femoral canal dimension was 18.5 ± 2.9 mm (range 16.2 to 22.7 mm), and the average cortical index was 0.37 ± 0.23 (range 0.21 to 0.48).

### Operative technique

THA surgery was performed under general or spinal anesthesia. In all cases, the posterolateral approach was used, with the patient placed on the contralateral side. One centimeter posteriorly from the tip of the greater trochanter, a skin incision, extended straight for approximately 12 cm, was performed. The gluteus medius was retracted, and the short extrarotators were isolated and detached. The insertion of the quadratus was spared if possible. Capsulotomy was performed, and the hip was dislocated by pulling, flexing, and internally rotating the lower limb. The femoral neck was resected at a designed level and the femoral head was removed. The primitive acetabulum was reamed, and al uncemented, press-fit cup was applied in or close to the anatomical position. Metal-polyethylene coupling was used in 20 hips, while in 8 hips ceramic–ceramic coupling was used. The medullary canal was reamed using tapered reamers of progressively increasing size until the planned size was achieved and until the resistance of the reamer against the femur cortex was felt. Then, a Wagner SL prosthesis uncemented stem was inserted by guiding it with its appropriate device. After the hip has been reduced, stability and range of motion were assessed, and limb length checked in comparison to the contralateral limb.

On the first day of postoperative care, the drainage was removed and physiotherapy was begun. The physical therapy was aided by a physiotherapist to make passive-active movements of the operated limb, to stand up and walk with the use of a walking frame. Initially, a dragged load on the operated limb was allowed, and then partial weight-bearing was allowed with the use of two crutches. The patients were checked clinically and radiographically 3, 6, and 12 months after surgery and yearly thereafter until an average follow-up of 72 ± 8.5 months (range 57 to 85 months). Twelve months after surgery and at last available follow-up, the clinical outcome was rated using the Harris hip score. On radiographs, postoperative radiographs were evaluated to check for stem alignment, stem-to-canal fill, biological fixation, and subsidence. Other observations included stress shielding, osteolysis, and cortical hypertrophy. Stem alignment on the anteroposterior radiograph was defined as varus if the tip of the stem was lateral by > 2 mm to a perpendicular line drawn down the femoral shaft and as valgus if the tip was medial to this line by > 2 mm. Overall stem-to-canal fill percentage [16] was assessed from proximal to distal section for both the anteroposterior and lateral projections. Moreover, femoral component stability was determined using the criteria of Engh et al. [17] for bony ingrowth. The ingrowth was classified as bone-ingrown, stable fibrous ingrowth, and unstable. Subsidence was determined by comparing measurements between two serial radiographs as described by Pelligrini et al. [18].

All continuous data were expressed as the mean and standard deviation of the mean, and the paired T test was performed to compare preoperative and postoperative Harris hip scores; *P* < 0.05 was considered significant.

## Results

The mean follow-up period was 125 ± 10.5 months (range 96 to 156 months). Average Harris hip score was 46 ± 9 (range 39 to 62) preoperatively, 88 ± 8 (range 78 to 96) at 12 months after surgery, and 90 ± 9 (range 83 to 98) at last follow-up. Nineteen of the 25 patients (76.0%) achieved excellent HHS scores (> 90), 3 (12.0%) good (80–89), 2 (8.0%) fair, and 1 (4.0%) poor. One patient with a fair score had clinically significant thigh pain.

**Fig. 1:**
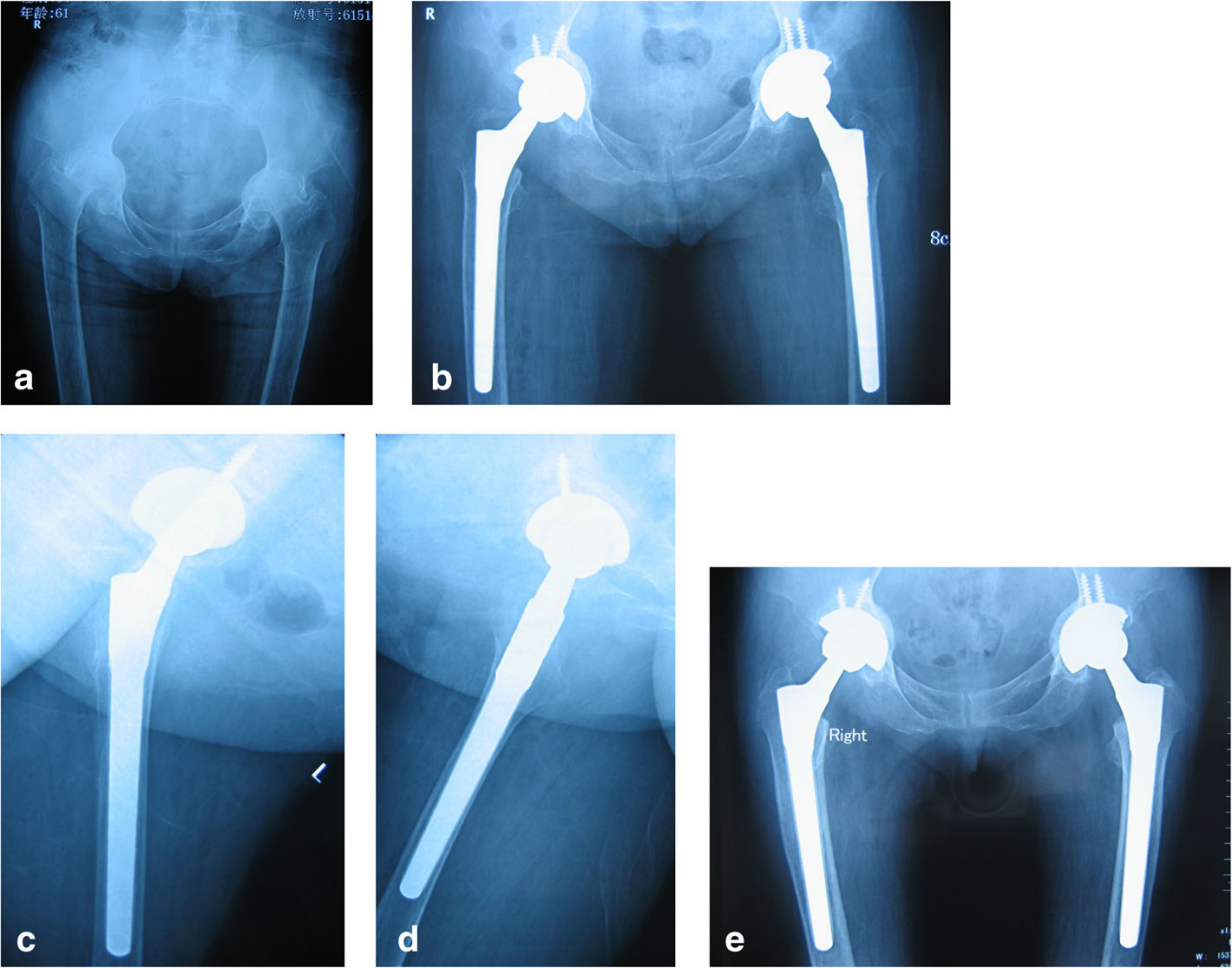
Radiographs of a 61-year-old woman with rheumatoid arthritis of both hips and Dorr type C bone. **a** Preoperative radiographs reveals acetabular protrusions of both hips and the wide straightened stovepipe intramedullary canals combined with widened isthmus and thin cortices. **b** Immediate postoperative anteroposterior radiographs demonstrating the canal filling of the inserted femoral component. **c**, **d** Frog-leg lateral radiograph in the left side and right side. **e** Postoperative radiograph of both hips obtained 3 years after surgery shows that stem is solidly fixed in a satisfactory position in both hips

**Fig. 2:**
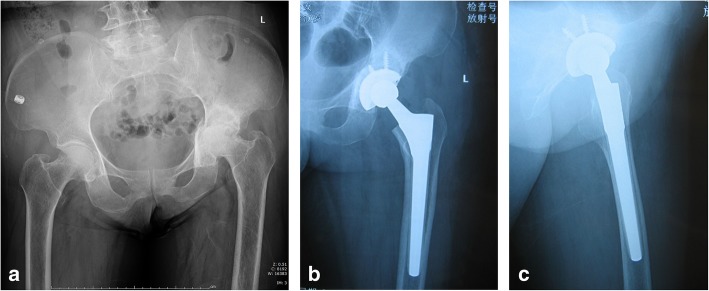
Radiographs of a 70-year-old woman with rheumatoid arthritis of the left hip. **a** Preoperative radiograph of left hip demonstrates the collapse of the left femoral head with Dorr type C femoral bone. **b** Immediate postoperative anteroposterior radiographs demonstrate the good canal filling of the Wagner SL stem. **c** Frog-leg lateral radiograph in left side

Femoral stem filling was assessed as percentages at three distinct levels: just below the lesser trochanter, at midstem, and 1 cm above the tip of the component on post-operative X-ray, which was calculated as SW (stem width)/ID (inner diameter of the femur) × 100 in millimeters. The mean proximal stem-to-canal fill percentages were 97% ± 2.1%, 95% ± 3.5%, and 88% ± 2.6%, respectively (anteroposterior view) and 92% ± 2.2%, 86% ± 1.9%, and 83% ± 2.5%, respectively (lateral view). Stem alignment revealed a varus position in 5 femurs (Fig. 1a–e) and a valgus position in 1; the other 19 hips exhibited neutral alignment (Fig. 2a–c).

Based on Engh classification [17], 23 hips had radiographic evidence of a bone ongrown prosthesis, and there was stable fibrous fixation in 2 hips. At the final follow-up (average, 72 ± 8.5 months), bone resorption and stress shielding were noticeable in 5 hips (grade 1 in 3 hips, grade 2 in 2 hips, as described by Engh [19]. Stem subsidence was present in three stems. Subsidence was measured using bone-prosthetic landmarks on comparison radiographs. The range of stem subsidence was 2 mm in two stem and 3 mm in one stem. No definite clinical or radiographic evidence of implant loosening was observed. None of the implants has been revised. Cortical hypertrophy of the femur was seen in 18 hips. This hypertrophy was observed most commonly in Gruen zones 3 and 5.

There was no intraoperative femur fracture and femoral perforation during the canal rasping or stem implanting. No early infection or wound healing problems occurred. One patient underwent a deep venous thrombosis that was adequately managed on postoperative care and resolved without sequelae. One dislocation occurred within 1 month postoperatively, treated with close reduction and a hip brace, without the need for further surgery, and no recurrence was observed.

## Discussion

The morphology of the proximal femur differs widely according to age, race, sex, and lifestyle [2, 3, 20]. Some pathologic factors may also affect the geometry of the femur, such as rheumatoid arthritis, atrophic osteoarthrosis, osteoporosis, and some metabolic bone diseases [21, 22]. Type C bone is found predominantly in women of older ages and lower body weight, and it has both structural and cellular compromise [1]. Cortices are thin with complete loss of the medial and posterior cortices resulting in a “stovepipe” [3] shape of the intramedullary canal. These alterations increase the difficulty of joint replacement and may also threaten the immediate fixation and long-term survival of the prosthetic implant [1, 3–6]. The poor bone quality with an enlarged femoral canal had led surgeons to prefer the use of cemented implants in this type of femurs [4, 6, 21] because some mismatches can be accommodated by the void-filling capacity of the cement layer in a cemented hip system [3]. However, fixation loosening of the cemented femoral component has remained the leading revision problem in relative young patients [23]. Meanwhile, fat embolism, pulmonary microemboli, and cardiac arrest associated with cementing are still potential threats to older patients [24, 25], because the large amount of cement is necessary for filling the widened canal in type C bone. With the current advance in prosthetic design, cementless fixation of the femoral stem has proven to be durable and predictable in total hip arthroplasty [4–6, 8–10]. More and more cementless prostheses have been successfully used in particular situations, such as elder patients with poor bone stock [10, 12, 26], abnormal shape of proximal femur [8], and also in revision surgery [11, 27–29]. Although the results of conventional large-diameter or tapered cementless stems ware generally good in the patients with type C bone [12, 27, 30], the degree of stem-to-canal fill and the parameters of cortical index and isthmus width which indicated the extremely wide stovepipe canals have seldom described in these literatures [4–6, 12, 30, 31], and few considers the Wagner SL stem as a possibility in primary total hip arthroplasty for type C bone.

In cementless total hip arthroplasty, femoral prosthetic design, cross-sectional area of the stem, proximal geometry of the femoral canal, as well as surgical techniques can all affect the degree of the stem fill [32]. Many kinds of cementless prostheses have been developed to improve bone-implant fit and promote biological fixation [3, 8, 11, 16, 18, 22, 26, 31]. However, there is currently no consensus regarding the ideal stem design to enhance various features such as survivorship and implant fit [16]. The optimal femoral cementless stem should occupy a close geometric matching between the femoral stems and proximal femoral canal to achieve accurate endosteal stem fit [3, 8, 16]. The type C bones often present a correspondingly straightened femoral canal profiled with a wide isthmus and thin cortex [3, 22], and the enlarged isthmus loses its metaphyseal hoop stress for the most primary taper stems [2]. Meanwhile, anatomically shaped stem and distally fixed cylindrical stems that designed with curve to fit the antecurvation of the femur [17] are not favorable to a wide, straightened canal in type C bone. In fact, most primary conventional implants cannot provide a close geometric matching to the extremely stovepipe canals [2–6, 31]. In an effort to avoid this mismatch between conventional cementless stems and compromised endosteal geometry in type C bones, a new short, metaphyseal-fitting anatomic cementless femoral stem was developed recently [33, 34]. However, a potential concern with the use of this type of short femoral component, particularly in elder patients with type C bone, is the loss of stability of the component and failure of osseous ingrowth [33].

In 1987, Wagner [11, 35] developed a straight, long-stemmed femoral component that that is designed for revision. The Wagner self-locking stem was the first fluted, tapered stem designed to bypass the areas of proximal bone loss and secure uncemented fixation in the remaining distal, well-preserved femur [28]. In his first reports, Wagner [11] indicated that the self-locking revision stem was used mainly in patients with large defects of the proximal femur in revision surgery. The cylindrical tapered design with raised ridges also increases the initial rotational stability of the prosthesis in revision hip surgery [35, 36]. Similar to some authors [27–29], we began to use the Wagner SL stem only in revision arthroplasties, and then we found that the revision femoral cavities often present a trumpet shape with the wide diaphyseal canal after the femoral prostheses were removed. It is interesting that the third-generation Wagner SL prosthesis was designed to a long-straight stem with a cylindrical tapered shape which had a close apposition to the stovepipe femoral canal. So we broadened the use of Wagner SL prostheses to the primary total hip arthroplasty in type C bones. In our practice, based on the long-straight cylindrical stem with a taper angle of 2°, the third-generation Wagner SL stem offers a close geometrical matching to the wide stovepipe femoral canal and allow a perfect fit and fill along the endosteal cortex from the metaphysis to diaphysis in type C femoral bone. The eight raised ridges provide rotational stability in the femoral canal and its full grit-blasted rough titanium surface also provides long-term biological fixation [28, 29]. Therefore, the Wagner SL stem is available in lengths of 190 to 305 mm and in diameters of 14 to 25 mm [12], so the Wagner SL implants systems currently provide stems in a wide range of sizes to allow proper sizing with the extremely wide stovepipe femoral canals in type C bone.

We used Wagner SL stem to perform primary total hip arthroplasties and achieved good short-term clinical and imaging results in patients with type C bone. According to HHS score, 89.3% of our group had good and excellent results. Despite the frequent occurrence of dislocations and stem subsidence of Wagner SL stem in revision surgery [27–29], our radiographic evaluation at 4–8 years follow-up showed good osteointegration and less subsidence of the stem in this series patients. We believe this phenomenon is due to the good endosteal stem fit and fill in type C femoral bone, meanwhile, beside the tapered shape of the new Wagner stem may improve the tendency to self-limit subsidence [36], the eight length-wise raised ridges can achieve stable anchorage in relative good bone stock in type C femoral bone rather than the poor bone stock in former prosthetic stem bed. However, the patients with type C bone always present the weak bone or thin cortices, meticulous surgical technique must be taken to avoid fracture or femoral perforation during reaming and implanting. In our experience, reaming is more exact when using a machine than doing reaming by hand.

This study has certain limitations. First, it was retrospective in nature and it included a relatively small series. Second, the duration of follow-up was short and was thus insufficient to allow conclusions to be drawn, because most prostheses exhibit good results at 5 to 7 years after the operation. However, there is strong evidence that early stability (< 2 years) of a cementless femoral stem is a good predictor of good late clinical results [37, 38]. Third, it represents two surgeon’s experience, and we also have less experience for use of this stem in particular type C bone patients with extremely weak bone or thin-walled cortices (cortical index of ≤ 0.2). However, we believe it provides insight into the performance of this prosthesis in a selected patient population with lesser quality bone in type C bone. Finally, we performed no interobserver variability studies of the radiographic to confirm the measurements by the single observer, and this may lead to bias in interpreting the radiographic in turn leading to errors of either underestimation or overestimation.

## Conclusions

In summary, the third generation cementless Wagner SL stem, designed with a long-straight cylindrical tapered geometry, offers a significantly good canal fit in type C femoral bone. We find that the cementless Wagner SL stems can achieve reliable stability by close apposition of the stem and extreme wide stovepipe femoral canal from metaphysis to diaphysis, both in terms of stem-to-canal fill percentage. Further long follow-up is needed to confirm these short-term results.

## Data Availability

The dataset supporting this article is available upon request; please contact the corresponding author.

## Abbreviations

THA: Total hip arthroplasty
Wagner SL stem: Wagner self-locking stem
